# Sand cat swarm optimization-based feedback controller design for nonlinear systems

**DOI:** 10.1016/j.heliyon.2023.e13885

**Published:** 2023-02-24

**Authors:** Vahid Tavakol Aghaei, Amir SeyyedAbbasi, Jawad Rasheed, Adnan M. Abu-Mahfouz

**Affiliations:** a Istinye University, Faculty of Engineering and Natural Sciences, Electrical and Electronics Engineering, Istanbul, Turkiye; b Istinye University, Faculty of Engineering and Natural Sciences, Software Engineering, Istanbul, Turkiye; c Department of Software Engineering, Istanbul Nişantaşı University, 34398 Istanbul, Turkiye; d Council for Scientific and Industrial Research (CSIR), Pretoria 0184, South Africa; e Department of Electrical and Electronic Engineering Science, University of Johannesburg, Johannesburg 2006, South Africa

**Keywords:** State feedback control, Nonlinear systems, Trajectory control, Metaheuristic algorithms, Sand cat swarm optimization (SCSO)

## Abstract

The control of the open loop unstable systems with nonlinear structure is challenging work. In this paper, for the first time, we present a sand cat swarm optimization (SCSO) algorithm-based state feedback controller design for open-loop unstable systems. The SCSO algorithm is a newly proposed metaheuristic algorithm with an easy-to-implement structure that can efficiently find the optimal solution for optimization problems. The proposed SCSO-based state feedback controller can successfully optimize the control parameters with efficient convergence curve speed. In order to show the performance of the proposed method, three different nonlinear control systems such as an Inverted pendulum, a Furuta pendulum, and an Acrobat robot arm are considered. The control and optimization performances of the proposed SCSO algorithm are compared with well-known metaheuristic algorithms. The simulation results show that the proposed control method can either outperform the compared metaheuristic-based algorithms or have competitive results.

## 1 Introduction

There has been extensive study of the open-loop unstable benchmarks such as inverted pendulums in modern control. Its nonlinear and underactuated yet simple structure makes it an interesting and challenging problem for the control engineers to handle. There are a number of robots, spacecraft, and marine vessels that can be represented by their models, and as a result, they can ignite the curiosity of the researchers and experts in the field of nonlinear control [29], [45]. From the structural point of view, they can be categorized as pendulum carts which can move along the *x*-axis with a stick installed to the pivot point of the cart, a pendulum that can have motions on both *x* and *y* horizons like *Furuta* pendulum, and a spatial three-dimensional pendulum capable of moving in x−y−z directions [41]. To evaluate the control performance of the inverted pendulums there exist numerous linear and nonlinear control strategies such as proportional integral derivative (PID), state-feedback controller, fuzzy Q-learning, sliding mode controller, type-2 fuzzy controller, Lyapunov-based and model predictive path integral control [2], [11], [18], [25], [27], [36], [40]. Among these control strategies, PID can be considered as the simplest one with three parameters to be tuned for optimal performance, but when it comes to controlling nonlinear complicated systems traditional PID controllers cannot perform well enough [17]. To conquer this, some other classical methods like linear quadratic regulator (LQR) and state feedback can be beneficial [3].

One of the main challenges for these intelligent and traditional control techniques is associated with the parameter tuning involved in their structure. Usually, based on the operator's knowledge and experience, these parameters are tuned (empirical gain tuning). For various control features such as good and fast transient response, reduced overshoots, settling times, and steady-state errors an optimal solution is exceedingly challenging to obtain. It is possible to think of this parameter tuning issue as an optimization problem in which a variety of nature-inspired and evolutionary algorithms can play a vital role in solving them.

In the last decades, many metaheuristic algorithms have been presented. The real-world problem with different dimensions is being resolved by the metaheuristic algorithms. The metaheuristic algorithms have the ability to find the best or near optimum solution. Based on the No-Free-Lunch (NFL) [44] there is no algorithm capable of finding all possible optimization problems' solutions. There exist many metaheuristic algorithms, however, they can be distinguished in terms of hindering them in trapping local optimum, convergence speed. Also, there are some other metrics to evaluate each metaheuristic algorithm's performance. Most newly proposed metaheuristic algorithms try to find the near-optimum solution with new inspirational rules [43]. The main objective of these algorithms is the trade-off between two phases: exploration and exploitation.

**Figure 1:**
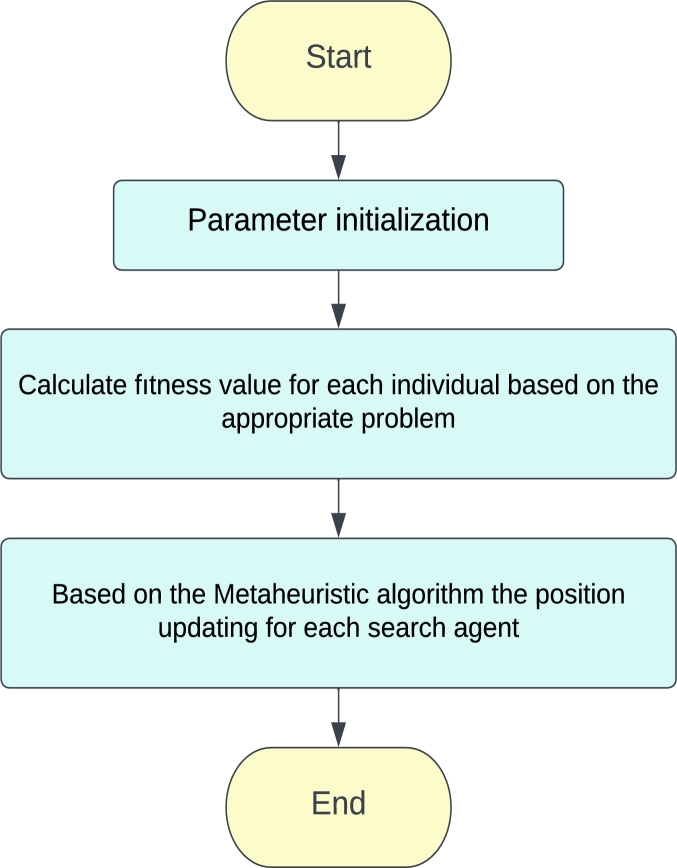
Metaheuristic algorithms mechanism.

Generally, the metaheuristic algorithms are divided into, evolution-based, physics-based, and swarm intelligence methods [32]. The evolution-based algorithms (EA) are inspired by the evolutionary rules in nature. The genetic algorithm (GA) [8], differential evolution (DE) [28], and Bio-geography-based optimizer (BBO) [21] are among the algorithms in the EA category. The physics-based algorithms originated from biological or physical phenomena. The gravitational search algorithm (GSA) [30], Big-Bang Big-Crunch (BBBC) [6], and black hole (BH) algorithms [9] are imitated by physical events. The swarm intelligence (SI) algorithms are inspired by the social behaviors of humans. Particle swarm optimization (PSO) [24], artificial bee colony (ABC) [15], ant colony optimization (ACO) [5], sand cat swarm optimization (SCSO) [33], grey wolf optimization (GWO) [23], and whale optimization algorithm (WOA) [22] belong to the SI category. The functionality of the metaheuristic algorithms is given in Fig. 1

In the context of classical control, there are gain-tuning methods for controllers based on various optimization algorithms. In the work done by [26], PID gains are calculated for an inductive system with a linear mathematical model in which the GA's fitness function is formed based on the settling time, rise time, and overshoot of the time response of the system. A hybrid particle swarm optimization (PSO) and differential evolution (DE) algorithm has been applied to a linearized tank-liquid level model to obtain the optimal PID gains to control the system in [24]. In the domain of modern control, PSO is used to design a linear quadratic regulator (LQR) for a three-phase inverter [38]. Big-Bang Big-Crunch (BBBC) is employed to optimize the PID and sliding mode controller gains in controlling the nonlinear model of a spatial inverted pendulum [41], [42]. Whale optimization algorithm (WOA) [34] is used to calculate the state feedback controller gains for a linear model of a thermal system. For the state feedback controller design, grey wolf optimization (GWO) is also applied to the control problem of a permanent magnet machine [35]. In the context of nonlinear systems [1] has used the Improved cuckoo optimization algorithm to solve the system of the nonlinear equations.

In this study, to learn and optimize the gains of the state feedback controller for nonlinear unstable systems of *inverted pendulum*, *Furuta pendulum*, and *Acrobat robot* for the first time the sand cat swarm optimization (SCSO) is proposed. Most recently, the SCSO as a novel nature-based algorithm is developed in [33]. The SCSO algorithm has several advantages over other metaheuristic algorithms, such as the exploration versus exploitation trade-off, the efficient performance in the convergence curve, and ease of implementation on different problems. Therefore, the SCSO method has been preferred and employed in some engineering problems [12], [13], [14], [20], [31]. Regardless of its recent successful performances, its applicability in real-time control problems of complex nonlinear systems has not been investigated yet. To fill this gap and benefit from the above-mentioned advantages of the SCSO, we apply it to the control engineering domain.

In this paper the contributions motivated by the need for novel and efficient optimization algorithms for developing optimal controllers in engineering problems can be summarized as follows:

1. **A lightweight yet powerful algorithm**
  A new state feedback controller design based on SCSO algorithm is proposed for the nonlinear unstable systems, reducing the tuning difficulties for optimal gains. The justification for hiring SCSO is its algorithmic simplicity owning to the fact that it has few parameters, making it easy to implement.
2. **Applications in real-world problems**
  The proof of the convergence of the SCSO algorithm is shown via various simulations when applied to highly nonlinear complex systems, which confirms the effectiveness of this algorithm in real-world engineering problems.
3. **Numerous comparisons**
  To verify the validity of the proposed SCSO-based control design, its performance has been compared with well-known optimization algorithms such as PSO, BBBC, WOA, and GWO in which it exhibited either better or close performance.

The rest of the paper is planned as follows. The proposed SCSO-based control algorithm and the physical model of the systems are presented in Section 2 as fundamentals. The simulation result and analysis are given in Section 3. Finally, the conclusions and future observations are written in Section 4.

## 2 Fundamentals

In the first part of this section, a general overview of the SCSO optimization algorithm will be provided. In the second part, we consider the equations of motions of three nonlinear systems and their control performance will be examined using some well-known metaheuristic algorithms. Finally, the proposed SCSO-based control algorithm will be explained.

### 2.1 The metaheuristic SCSO algorithm

The sand cat swarm optimization (SCSO) algorithm is inspired by the sand cat behaviors in nature [33]. The SCSO algorithm mimics the search and hunting mechanism of this animal. The sand cat has some specific characteristics in comparison to the domestic cats such as the ability to detect the low frequency noises, live in the harsh environment of deserts, and own specific methods for hunting. There are not any differences in the appearance of both cat types, but the sand cat has a high density of fur in the palms and soles. As mentioned earlier, the sand cat has a special method in the foraging and hunting mechanism. They predict the location of the prey according to their extraordinary ability in detecting low-frequency noises. The main characteristic of the sand cat is to hear noises with frequencies below 2kHz. The SCSO algorithm tries to find a solution close to the optimum. Based on the NFL theorem [44], one cannot find a metaheuristic algorithm capable of finding an optimum solution to each individual problem. Among the available metaheuristic algorithms, however, the SCSO algorithm has been shown to be effective with reliable performance in most of the optimization problems.

For implementing the SCSO algorithm, the first step is the initialization of the parameter search space. This step is similar to all population-based metaheuristic algorithms. The search space is populated randomly between the lower and upper boundaries of the parameters according to the limitations of the given optimization problem. The assigned parameter ranges determine the borders of the search space. In each iteration of the algorithm, the SCSO algorithm controls the boundaries of each parameter in the search space. The number of rows in the search space is equal to the number of the search agents and the number of columns representing the number of the dimensions of the problem. The randomly populated search space includes a list of candidate solutions that the metaheuristic algorithm should update and finally find a solution near the optimum value from the search space. For this and to evaluate the candidate solutions, a well-defined fitness function (cost) is required for each specific optimization problem. Based on the problem's goal, which could be either a maximization or minimization one, the metaheuristic algorithm tries to guide the search mechanism towards the optimum solution. In the next step, the search agents perform a continuous exploration within the search space to update their positions and move towards the regions where the optimal solution is placed, (reaching near to the prey's location). Here, the search and hunting mechanism of each metaheuristic algorithm is different.

In the SCSO algorithm, the searching for prey is empowered by each sand cat's ability in the low-frequency noise emission. The sensitivity range (*R*) of each search agent is predefined between 2 to 0. The $\overset{\to }{r_{G}}$ parameter indicates the general sensitivity range which decreases from 2 to 0 according to Equations. (1), (2), (3). In the equation (1) the $S_{M}$, which is associated with the sand-cat's ability to catch low-frequencies below 2 KHz, is assumed to be 2, $iter_{c}$ is the current iteration number, and $iter_{max}$ is the iterations maximum value. $\overset{\to }{X_{c}}$ indicates the current position, $\overset{\to }{X_{b}}$ indicates the best position, and $\overset{\to }{X_{rnd}}$ is the random position according to Equations (4), (5a), (5b).

In the SCSO algorithm, each search agent moves in a circular manner, in this way to move in different directions to search for possible global solutions, the random angle *α* between 0 to 360 is considered as a cos⁡(α) function. The main structural equations of the SCSO algorithm are given in Equation (6).

(1) $$\overset{\to }{r_{G}}=s_{M}-\left(\frac{s_{M}\times iter_{c}}{iter_{max}}\right)$$

(2) $$R=2\times \overset{\to }{r_{G}}\times rand(0,1)-\overset{\to }{r_{G}}$$

(3) $$\overset{\to }{r}=\overset{\to }{r_{G}}\times rand(0,1)$$

(4) $$\overset{\to }{X}(t+1)=\overset{\to }{r}\cdot \left(\overset{\to }{X_{b}}(t)-rand(0,1)\cdot \overset{\to }{X_{c}}(t)\right)$$

(5a) $$\overset{\to }{X_{rnd}}=\left|rand(0,1)\cdot \overset{\to }{X_{b}}(t)-\overset{\to }{X_{c}}(t)\right|$$

(5b) $$\overset{\to }{X}(t+1)=\overset{\to }{X_{b}}(t)-\overset{\to }{r}\cdot \overset{\to }{X_{rnd}}\cdot \cos(\alpha )$$

(6) $$\overset{\to }{X}(t+1)=\left\{ \begin{array}{cc} \overset{\to }{r}\cdot \left(\overset{\to }{X_{b}}(t)-rand(0,1)\cdot \overset{\to }{X_{c}(t)}\right) & \left|R\right|>1\\ \overset{\to }{X_{b}}(t)-\overset{\to }{r}\cdot \overset{\to }{X_{rnd}}\cdot \cos(\alpha ) & \left|R\right|\leq 1 \end{array}\right.$$

### 2.2 Physical systems model

To show the performance of the optimization algorithms we apply them to three different nonlinear systems; Inverted pendulum, Furuta pendulum, and Acrobat Robot. In the following, the mathematical formulations of the systems along with their parameters and states will be given.

#### 2.2.1 Inverted pendulum

The inverted pendulum system in modern control is a good example of a benchmark experiment for an open-loop unstable system which is being considered for different linear and nonlinear control applications. Its working principle generally is based on a moving cart over a rail with a stick (pendulum) attached to it. The control objective here is to stabilize the pendulum in a vertical position by applying desired control signals to the cart, considering the limitations on the position of the cart as well as the angle of the pendulum with respect to the vertical position. The continuous state space of the inverted pendulum is composed of four state variables as $X=[\phi \;\overset{˙}{\phi }\;p\;\overset{˙}{p}]$ which stand for pendulum's angle and its angular velocity, cart position and cart velocity, respectively. The control objective is to design a suitable feedback controller for this system capable of stabilizing the pendulum's angle in the vertical position as well as bringing the cart position to the origin. The dynamical equations of the system are given in Equations (7a), (7b) as shown by [4]

(7a) $$\overset{¨}{\phi }=\frac{-3ml{\overset{˙}{\phi }}^{2}\sin\phi \cos\phi +6(M+m)g\sin\phi -6(u-b\overset{˙}{\phi })\cos\phi }{4l(M+m)-3ml\cos\phi }$$

(7b) $$\overset{¨}{p}=\frac{-2ml{\overset{˙}{\phi }}^{2}\sin\phi +3mg\sin\phi \cos\phi +4u-4b\overset{˙}{\phi }}{4(M+m)-3m\cos\phi }$$

where the model parameters of the system are summarized in Table 1. For each state, a stabilizing feedback gain is learned by the optimization algorithms. To have a fair comparison between the metaheuristic algorithms, the same quadratic cost function is used which encompasses the information regarding the state variables and the generated control signal. This quadratic function is defined as:

(8) $$J(X,u)=X^{T}QX+R^{T}uR$$

with a positive definite weight matrix $Q_{4\times 4}$ defined as diag([10101010]) and a positive penalty value R=0.1 for the control signal *u*. For this cost function to have a global minimum, the matrices Q and R have to be positive definite. A matrix is positive definite if it is symmetric and all its eigenvalues are positive. If these matrices are positive definite, then the function *J* is a strictly convex function. A strictly convex function has a unique global minimum, which can be found by taking the derivative of the function and setting it to zero. This ensures that the optimization algorithm can converge to the optimal solution. It is also important to check that the domain of X and *u* is closed and bounded.

**Table 1:** Inverted pendulum's model parameters.

*M*	Cart mass (kg)	0.5
*m*	Stick mass (kg)	0.5
*l*	Stick length (*m*)	0.6
*b*	Friction constant (*N*/*m* × *s*)	0.1
*g*	Gravity (*m*/*s*^2^)	9.81

#### 2.2.2 Furuta pendulum

The Furuta pendulum is an underactuated nonlinear system whose structure is composed of a rotating arm with a pendulum attached to it. For this system also, we consider four continuous states consisting of the arm angle, arm angular velocity, pendulum angle, and its angular velocity represented as $X=[\theta_{a}\;{\overset{˙}{\theta }}_{a}\;\theta_{p}\;{\overset{˙}{\theta }}_{p}]$. The governing equations of the Furuta pendulum with respect to the arm and pendulum angles are represented in [7] and given as follows:

(9) $${\overset{¨}{\theta }}_{a}=\frac{1}{D}[(J_{1}+m_{1}l_{1}^{2})\tau -(J_{1}+m_{1}l_{1}^{2})m_{1}l_{1}^{2}\sin(2\theta_{p}){\overset{˙}{\theta }}_{a}{\overset{˙}{\theta }}_{p}-0.5m_{1}^{2}l_{1}^{3}L_{0}\cos\theta_{p}\sin(2\theta_{p}){\overset{˙}{\theta }}_{a}^{2}+(J_{1}+m_{1}l_{1}^{2})m_{1}l_{1}L_{0}\sin\theta_{p}{\overset{˙}{\theta }}_{p}^{2}-m_{1}l_{1}^{2}L_{0}g\cos\theta_{p}\sin\theta_{p}]{\overset{¨}{\theta }}_{p}=\frac{1}{D}[-(m_{1}l_{1}L_{0}\cos\theta_{p})\tau -m_{1}^{2}l_{1}^{2}L_{0}^{2}\sin\theta_{p}\cos\theta_{p}{\overset{˙}{\theta }}_{p}^{2}+m_{1}l_{1}^{2}\sin(2\theta_{p}){\overset{˙}{\theta }}_{a}(m_{1}l_{1}L_{0}\cos\theta_{p}{\overset{˙}{\theta }}_{p}+0.5(I_{0}+m_{1}L_{0}^{2}+l_{1}^{2}\sin^{2}\theta_{p}){\overset{˙}{\theta }}_{a})+(I_{0}+m_{1}L_{0}^{2}+l_{1}^{2}\sin^{2}\theta_{p})m_{1}l_{1}g\sin\theta_{p}]$$

where D is obtained by Equation (10)

(10) $$D=(I_{0}+m_{1}l_{1}^{2}\sin^{2}\theta_{p})(J_{1}+m_{1}l_{1}^{2})+J_{1}m_{1}L_{0}^{2}+m_{1}^{2}l_{1}^{2}L_{0}^{2}\sin^{2}\theta_{p}$$

The parameters of the Furuta pendulum model in Equation (9) are defined according to Table 2.

**Table 2:** Furuta pendulum's model parameters.

*m*_1_	pendulum mass (kg)	5.38 × 10^−2^
*L*_0_	Arm length (m)	0.215
*g*	Gravity (m/s^2^)	9.81
*l*_1_	Distance to pendulum's center of gravity	0.113
*J*_1_	pendulum Inertia	1.98 × 10^−4^
*I*_0_	Arm Inertia	1.75 × 10^−2^

#### 2.2.3 Acrobat robot

**Figure 2:**
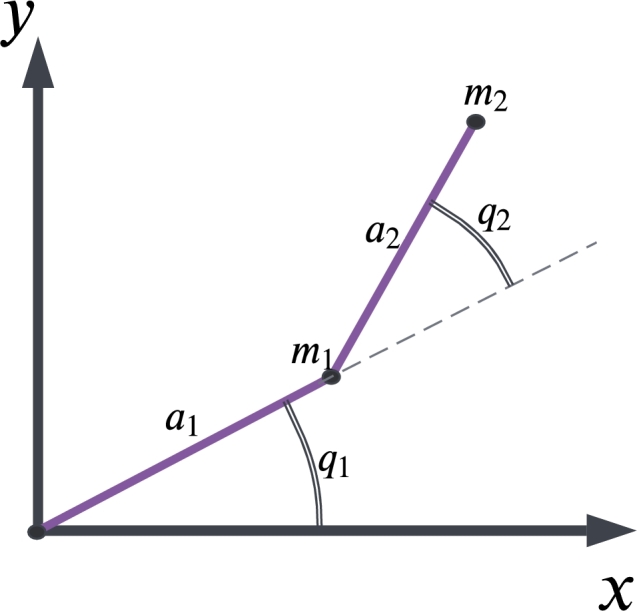
Mechanism of the acrobat robot.

A simple structure of the 2-link robot known also as the Acrobat robot is given in Fig. 2, where it is used to obtain the dynamical equations of the robot, assuming that the mass of the links is $m_{1}$ and $m_{2}$ to be equal to 1kg, their lengths are $a_{1}$ and $a_{2}$ equal to 1m, joint angles of the links as $q_{1}$ and $q_{2}$. These links are controlled by two motors with torques $\tau_{1}$ and $\tau_{2}$. Using the *Lagrangian* formulation, the general form of the acrobat robot's dynamics can be written as given in Equation (11)

(11) $$M(q)\overset{¨}{q}+V(q,\overset{˙}{q})+G(q)=\tau$$

where *M*, *V*, and *G* stand for inertia matrix, Coriolis, and gravity vectors, respectively. One can extract the dynamics of the acrobat robot as shown in Equation (12), which are elaborately covered in [19].

(12) $$\left[\begin{array}{cc} (m_{1}+m_{2})a_{1}^{2}+m_{2}a_{2}^{2}+2m_{2}a_{1}a_{2}\cos q_{2} & m_{2}a_{2}^{2}+m_{2}a_{1}a_{2}\cos q_{2}\\ m_{2}a_{2}^{2}+m_{2}a_{1}a_{2}\cos q_{2} & m_{2}a_{2}^{2} \end{array}\right]\left[\begin{array}{c} {\overset{¨}{q}}_{1}\\ {\overset{¨}{q}}_{2} \end{array}\right]+\left[\begin{array}{c} -m_{2}a_{1}a_{2}(2{\overset{˙}{q}}_{1}{\overset{˙}{q}}_{2}+{\overset{˙}{q}}_{2}^{2})\sin q_{2}\\ m_{2}a_{1}a_{2}{\overset{˙}{q}}_{1}^{2}\sin q_{2} \end{array}\right]+\left[\begin{array}{c} \left(m_{1}+m_{2})ga_{1}\cos q_{1}+m_{2}ga_{2}\cos(q_{1}+q_{2}\right)\\ m_{2}ga_{2}\cos(q_{1}+q_{2}) \end{array}\right]\left[\begin{array}{c} \tau_{1}\\ \tau_{2} \end{array}\right]$$

To control the acrobat robot, Proportional-Derivative Computed Torque (PD-CT) is used and its working principle is based on the feedback linearization [37]. For this, first, an error trajectory is defined as Equation (13):

(13) $$e(t)=q_{d}(t)-q(t)$$

The nonlinear controller which ensures tracking the desired motion trajectory $q_{d}(t)$ can be written as Equation (14):

(14) $$\tau =M(q)(\overset{¨}{q}-u)+N(q,\overset{˙}{q})$$

where $N(q,\overset{˙}{q})$ is composed of nonlinear terms defined by Equation (15):

(15) $$N(q,\overset{˙}{q})\equiv V(q,\overset{˙}{q})\overset{˙}{q}+F(\overset{˙}{q})+G(q)$$

with friction components represented by $F(\overset{˙}{q})$. Taking the control signal as a PD controller will yield the PD-CT controller as provided in Equation (16):

(16) $$\tau =M(q)(\overset{¨}{q}+k_{p}e+k_{d}\overset{˙}{e})+N(q,\overset{˙}{q})$$

**Figure 3:**
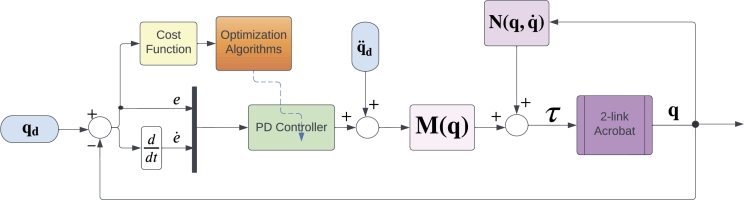
Block diagram representation of the PD-CT with the online optimization algorithms.

The structure of the resulting PD-CT control loop is given in Fig. 3, where the optimization algorithms are responsible for learning the controller gains. It is built with a multiloop structure, consisting of an inner nonlinear feedback linearization loop and an exterior unity-gain tracking loop. It is important to keep in mind that for each joint there is an outer loop. In this closed-loop control system, we have

$$q_{d}=[q_{d}\;\;{\overset{˙}{q}}_{d}]\;q=[q\;\;\overset{˙}{q}]$$

The desired trajectory is also assumed to be a sinusoidal one with a period of 2 and amplitude of 0.1 according to Equation (17):

(17) $$q_{d}=[0.1\sin(\pi t)\;\;\;0.1\cos(\pi t)]$$

We defined the cost function of the applied optimization algorithms considering the error trajectory as:

(18) $$J=e^{2}Q$$

where *Q* is a diagonal matrix as Q=diag([1010]);

### 2.3 SCSO-based controller design

**Figure 4:**
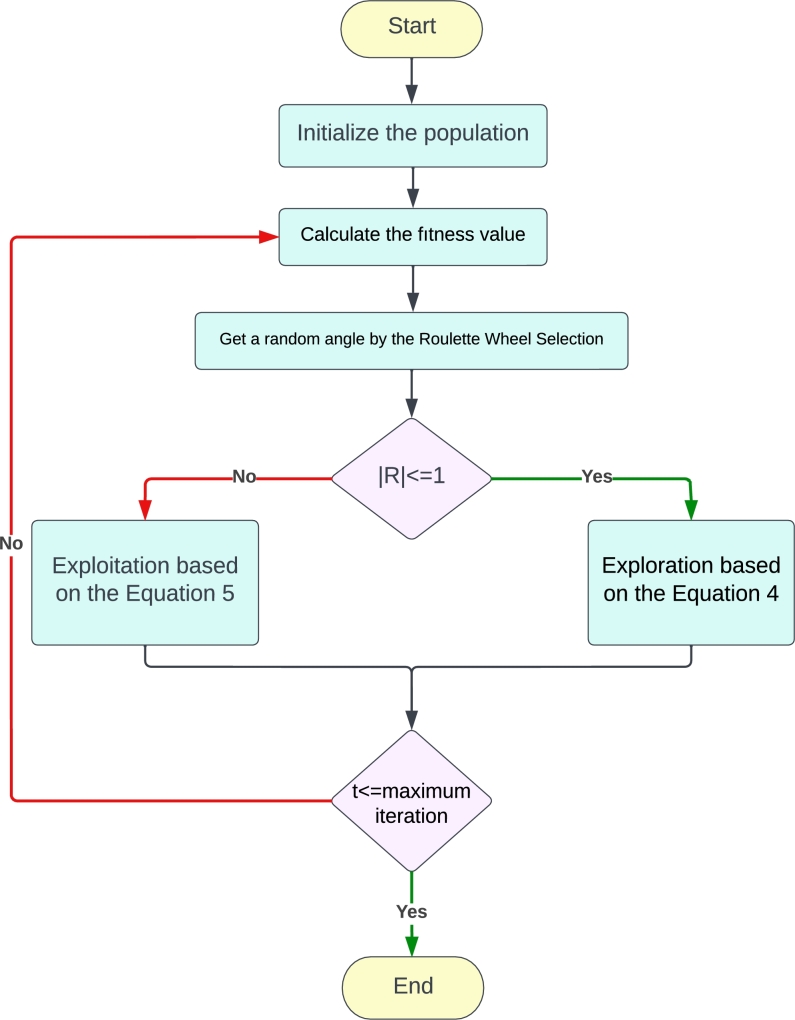
Flowchart for the SCSO algorithm.

The fundamental principle of operation of the SCSO method is to find the possible optimal solutions from a random search space (seeking for prey and attacking them). The objective of the algorithm based on the problem at hand can be either minimization or maximization of an appropriate cost function. Fig. 4 demonstrates SCSO's flowchart. According to this flowchart and based on the following steps, the optimal control parameters for the nonlinear unstable systems can be determined.

*Step 1*  The first step is to assign the initial parameters for the SCSO-based control algorithm as well as define the lower and upper bounds for the unknown parameters in the search space; For the inverted pendulum and Furuta pendulum, the parameters to be obtained are the gains of the state feedback controller, which for these systems we have in total 4 controller gains. For the acrobat robot, the PD gains will be obtained. Moreover, the number of search agents, and the maximum number of iterations should be specified. Then, the initialization of the random parameter space according to the boundary conditions can be done based on Equation (19):

(19) $$x_{0}=rand(dim,1)\times (ub_{\theta_{i}}-lb_{\theta_{i}})+lb_{\theta_{i}}$$

where *ub* and *lb* are the upper and lower boundaries for each $\theta_{i}$ parameter to be learned and *dim* is the dimension of each parameter.

*Step 2*  In step two, each search agent calculates the fitness value of the appropriate possible solution based on the available problem. For the inverted pendulum and Furuta pendulum, the relevant fitness function is given in Equation (8) and for the acrobat robot it is given in Equation (18).

*Step 3*  SCSO is initiated here in order to determine the optimum solution. On the basis of the fitness function, the best score and the best position of the optimum solution are determined. Accordingly, the SCSO algorithm updates the search agent positions based on Equation (1). It is worthwhile mentioning that selecting an appropriate performance index for the metaheuristic algorithm is crucial.

*Step 4*  As part of the SCSO algorithm, a predefined stopping criterion is assigned. In our case, the stopping criteria are met when the iteration maximum value is reached.

## 3 Simulation and result analysis

A metaheuristic algorithm typically produces different results depending on the values of its control parameters, and it is often found that different control parameters produce dissimilar results depending on their values. In this section first, the initialization of the compared metaheuristic algorithms such as GWO, WOA, PSO, and BBBC with the SCSO is presented. Then, they are applied to the nonlinear models of the inverted pendulum, Furuta pendulum, and the acrobat robot to learn a suitable state-feedback controller gain vector to stabilize and control them. Their control behavior is then illustrated as time response plots and the stabilizing gains and resulted cost values are provided in relevant tables. Finally, the section will be concluded with some discussions regarding the extracted observations from the results.

### 3.1 Parameter initialization

For all the considered meta-heuristic algorithms including the proposed SCSO algorithm, it is assumed that each one is run with a population size of 30 and a number of iterations of 500. The simulation configuration composed of the different initial parameters and specific parameter ranges for each meta-heuristic algorithm, is given in Table 3 To have a fair evaluative comparison, the environment in which all the experiments are conducted, is considered under the same conditions. All the simulations are implemented using a Core i7−5500U2.4 processor, 8 GB of RAM in the MATLAB 2020 environment.

**Table 3:** Initialization of the algorithms.

Algorithm	Parameter	Value
**SCSO**	Sensitivity Range (*r*_*G*_)	[0 2]
	Phase Control Range (R)	[−2*r*_*G*_ 2*r*_*G*_]

**GWO**	a linearly decreasing vector (*a*)	[0 2]
	*A* coefficient vector	[0 2]
	*C* coefficient vector	2 × *rand*(0,1)

**WOA**	*a* decreases linearly from 2 to 0	[0 2]
	*A* coefficient vector	[0 2]

**PSO**	Weight *w*	[0.4 0.9]
	Acceleration constants *c*_1_,*c*_2_	2

**BBBC**	Iteration parameter *N*_*g*_	30
	Space limiting parameter *β*	0.15
	Acceleration constant *c*_1_	[1 15]
	Acceleration constant *c*_2_	[2 20]

### 3.2 Result analysis

**Figure 5:**
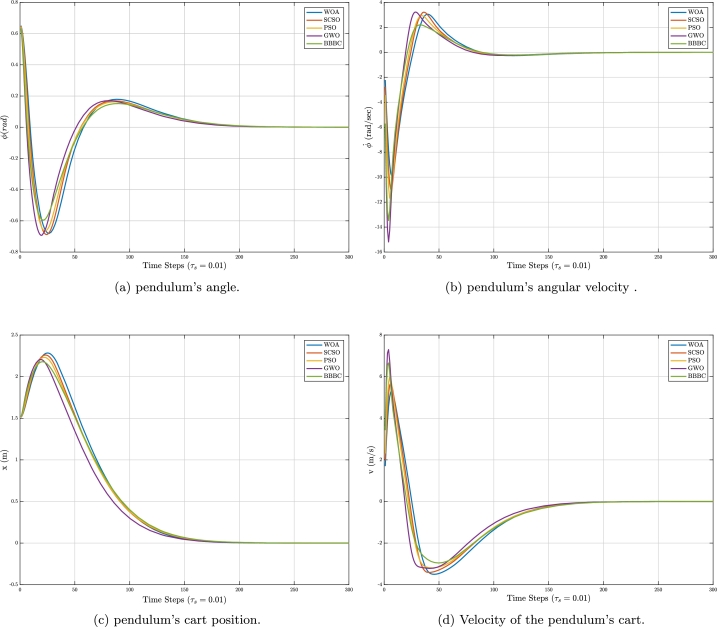
Time responses for the Inverted pendulum.

**Figure 6:**
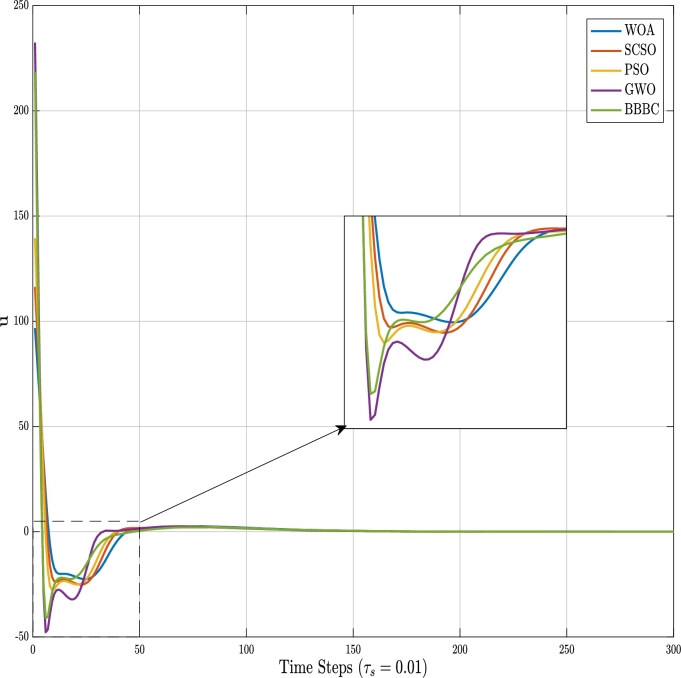
Control signals for the Inverted pendulum.

To show the effectiveness of the proposed SCSO parameter optimization for the controller design of physical systems, a comparison based on different metaheuristic algorithms such as PSO, BBBC, WOA, and GWO in three complex nonlinear systems as represented in Section 2.2, has been made. To control the Inverted pendulum and Furuta pendulum, we design two different full-state feedback controllers according to the number of states involved for each system. A state feedback controller is represented according to Equation (20):

(20) u=−kx

where *k* stands for a gain vector of the controller, which is responsible for stabilizing the pendulum angles, and *u* is the resulting control signal. Specifically for the Inverted pendulum, another control objective is to steer the system's cart position towards the origin from any given initial cart position, as well. The time response variations of the inverted pendulum for the obtained state vector by metaheuristic algorithms are given in Fig. 5, where the simulation time is chosen to be 10 sec with a sampling time of $\tau_{s}=0.01$ sec. It should be emphasized that for a more accurate portrayal of the plots, we showed only 3 seconds of the simulation in this figure. To obtain the time response plots of the system, an initial state is assigned as $X_{0}=[0.66\;(rad)\;\;0.3\;(rad/s)\;\;1.5\;(m)\;\;0.3\;(m/s)]$. Although according to Fig. 5, the performance of the applied meta-heuristic algorithms for stabilizing the states of the Inverted pendulum is very close to each other, we can discuss the performance of each algorithm according to the desired specifications of the system. Specifically, when considering Fig. 5a, in terms of the undershoots and overshoots of the angle deviations, BBBC demonstrates better performance. Among the algorithms, GWO decreased the rise time of the system's response, whereas WOA resulted in a greater rise time. Although PSO and SCSO exhibited similar performances, PSO seems to result in a less undershoot for the angle response. For the pendulum's cart position shown in Fig. 5c, the performance of GWO can be distinguished from the other algorithms, where it shows a reduced rise-time, settling-time, and overshoot. The behavior of the control signal for the Inverted pendulum, is also shown in Fig. 6. As observed from this figure, less initial control effort devoted to controlling the inverted pendulum belongs to WOA, SCSO, PSO, BBBC, and GWO, respectively (the less control effort, the better the controller design). Moreover, one can observe the velocity behavior of the pendulum's angle and the cart in Figs. 5b, 5d.

**Figure 7:**
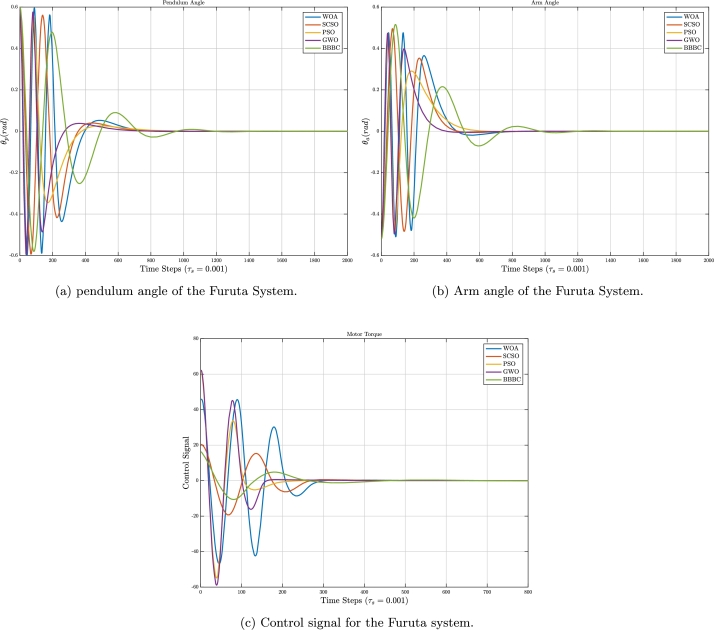
Time responses for the Furuta pendulum.

The simulation results of the Furuta pendulum are shown in Fig. 7. To have a clear and reliable comparison, specifically for Fig. 7a, assuming the starting time for the comparison is around 150, in terms of the overshoots, the PSO owns the first place by having the minimum value, SCSO and GWO are in the second place, and BBBC has the maximum value of the overshoot. In Fig. 7b, SCSO owns the second-best performance after GWO, in having a better settling time.

For hardware setup implementations, the behavior of the output control signals of the systems is crucial. When considering Fig. 7c, the control signal variations for GWO, WOA, and PSO are relatively large, and in real-world physical experiments depending on the limitations of the available actuators, they could not be realistic. Whereas, these variations for BBBC and SCSO are small, which could give better results in real-world experiments.

**Figure 8:**
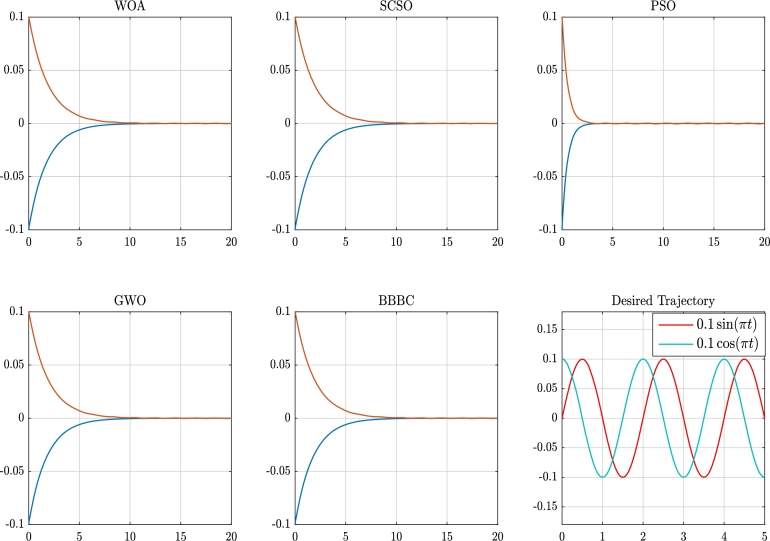
Trajectory error and the desired trajectory for the acrobat.

**Figure 9:**
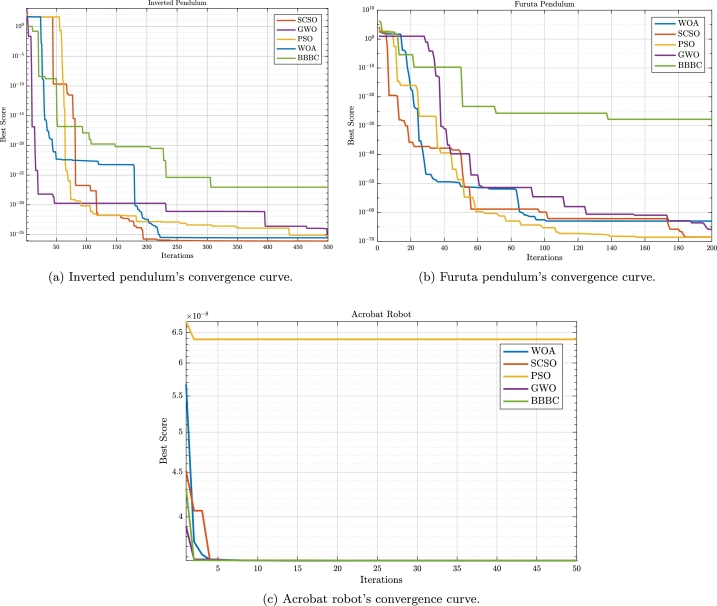
Convergence plots for the system models.

For the Acrobat robot, the resulted trajectory error plots, for the given sinusoidal inputs are given in Fig. 8. As can be seen, while PSO outperforms the other algorithms, SCSO, WOA, GWO, and BBBC show a similar trend in tracking the inputs. In terms of the algorithmic analysis we can discuss the convergence of the applied metaheuristic optimization methods according to Fig. 9 and the given Table 4, Table 5, Table 6, where for each system the obtained state feedback parameters and the resulting cost values, are given. According to our results, there are similar functional behaviors in all of these nonlinear system models as they are in the uni-modal fixed function models; the best or optimal solution for these types of functions can only have one global solution. One of the objectives of our study is to examine the metaheuristic algorithms' capacity for exploration and exploitation by the knowledge that finding a global optimum solution requires a good level of exploitation. The optimized gains for the pendulum's angle, angular velocity, the cart's position, and its linear velocity are given in Table 4 and represented by $k_{i}$ where i=1,…,4. The functionality of the implemented metaheuristic optimization algorithms is based on an appropriate cost function. The converged value of the cost function for each algorithm is also given in Table 4 which is calculated according to Equation (8). As it is obvious from this table, the cost value of the SCSO algorithm is better than the others.

**Table 4:** Optimized parameters and the converged cost function for the Inverted pendulum.

Algorithm	*k*_1_	*k*_2_	*k*_3_	*k*_4_	Cost
SCSO	−106.5173	−15.3428	−24.3197	−16.5111	**6.06*e* − 37**
GWO	−199.7924	−28.3381	−54.4326	−34.7815	1.78*e* − 36
WOA	−90.1191	−13.1692	−19.5456	−13.3067	1.42*e* − 34
PSO	−126.4230	−18.1780	−29.7044	−20.1036	1.38*e* − 35
BBBC	−195.7704	−28.2128	−47.3587	−32.1615	4.65*e* − 32

**Table 5:** Optimized parameters and the converged cost function for the Furuta.

Algorithm	*k*_1_	*k*_2_	*k*_3_	*k*_4_	Cost
SCSO	−30.9614	−8.6242	−60.8159	−7.3316	**3.14*e* − 69**
GWO	−102.1752	−27.9689	−192.2926	−23.1986	1.39*e* − 66
WOA	−98.7970	−23.3394	−161.9467	−19.3776	1.04*e* − 63
PSO	−101.0838	−27.7087	−191.1006	−23.0322	6.70*e* − 70
BBBC	−38.5966	−12.1674	−60.6569	−10.6516	1.73*e* − 28

**Table 6:** Optimized parameters and the converged cost function for the acrobat robot.

Algorithm	*k*_*p*_	*k*_*d*_	Cost
SCSO	117.3465	200	3.5629*e* − 08
GWO	117.84117	200	3.5636*e* − 08
WOA	117.5706	200	3.5634*e* − 08
PSO	200	107.1564	6.3848*e* − 08
BBBC	117.5924	−12.1674	3.5636*e* − 08

The obtained results of the Furuta pendulum, which is designed to rotate an arm attached to a pendulum, are also shown in Table 5. Similar to Table 4, there are gain parameters to be optimized, which should control the arm angle, its angular velocity, the pendulum angle, and its angular velocity. According to Table 5, the SCSO algorithm also provides better results in comparison to other algorithms for finding optimized parameters. For this system, the cost function values are obtained using Equation (8).

Table 6 shows the result that was obtained for the acrobat robot system model during the simulation process. In this system model, there are two gain parameters that control the angles shown in Fig. 2. According to the obtained results, the cost values are competitive. For this system, we have used the cost function given in Equation (18). It is worth noting that, except for BBBC, the other algorithms have crossed their parameter boundaries. Based on Fig. 9a, 9b, and 9c convergence curves of the metaheuristic algorithms are presented. As can be seen from this figure, the SCSO algorithm's convergence rate, in terms of keeping the trade-off between the exploration and exploitation phases, is satisfactory.

### 3.3 Discussions

Although in Section 3.2 we provide the time-response and convergence properties of the proposed SCSO control algorithm and showed its effectiveness when compared to some optimization algorithms, there still exist some inquiries that should be explored.

• In our simulations, we did not incorporate the effect of the disturbances, parameter uncertainties, and noises involved, and ideal conditions are taken. Actually, to design robust control and optimization algorithms that can be better integrated with real-world system models, the role of the disturbances and the uncertainties are indispensable.
• As the control algorithms for our systems, the classical state-feedback, and PD-CT are hired. Other sophisticated traditional and intelligent controllers could also have been used like a hybrid-neural network-based sliding mode control (SMC) [16], nonlinear SMC [39], and Fuzzy control [10].
• The obtained result for the robotic arm falls into the boundaries of the initial parameters except for the BBBC algorithm. This is because of the exploration-exploitation behavior of the metaheuristic algorithms. This can be overcome by modifying the range of the parameters defined. Assigning the bounds for the parameter spaces can be tricky and problem-specific, therefore it is crucial to have a firm grasp of the issue at hand before you start making assumptions.
• Based on the obtained result for each nonlinear system model, the metaheuristic algorithms get the optimum value as a cost function. However, the obtained optimum cost for the acrobat robot for all the algorithms is in a competitive manner, which shows the sensitivity and accuracy of the algorithms.

## 4 Conclusions and future observations

In this paper, for the first time, the SCSO-based state feedback controller is proposed and successfully applied to three nonlinear physical systems. In this direction, first, we explore the stabilization performance of the applied optimization-based control strategy in the Inverted pendulum and Furuta pendulum. Then the trajectory tracking control of an Acrobat robot is investigated. To put our methodology in context and demonstrate its efficiency, a thorough comparison has been made with some famous optimization algorithms such as BBBC, PSO, WOA, and GWO in terms of both control performance and convergence. The main tasks of the current paper can be pointed out in the following:

• The proposed SCSO-state feedback controller is implemented in the controller design for the nonlinear, unstable, and underactuated models of the Inverted pendulum and Furuta pendulum, where their control is challenging work. The resulting control can successfully stabilize the Inverted pendulum's angle and cart position. Similarly, the Furuta pendulum managed to stabilize both the pendulum and arm angles based on the designed controller.
• To guarantee the tracking error for given sinusoidal reference inputs to the Acrobat robot, an SCSO-based PD-CT controller is designed.
• The results of the simulations for both the convergence and control performance reveal the competitiveness of the proposed SCSO-based control design with some famous optimization algorithms. In relation to the time response features of the systems, we can infer that the SCSO-based control can even perform better than the tested optimization algorithms, or its performance is sufficient to match those of the competitors and achieve the intended control objectives.
• From the algorithmic point of view, SCSO inherits a simple structure and is easy to implement, which entitles it as a suitable candidate for real-world control and engineering problems. Moreover, it can ensure an accurate convergence by retaining a balance between exploration and exploitation.

As a future research path, we first intend to extend the optimization idea from the classical to the advanced intelligent control algorithms and apply the SCSO algorithm to learn the model of the fuzzy controllers which may demand a high-dimensional parameter optimization space. The next step is to investigate the robustness of the proposed SCSO algorithm in terms of disturbances, parameter uncertainties, and noises applied to the system model.

## Funding statement

This research did not receive any specific grant from funding agencies in the public, commercial, or not-for-profit sectors.

## CRediT authorship contribution statement

Vahid Tavakol Aghaei and Amir SeyyedAbbasi: Conceived and designed the experiments; Performed the experiments; Analyzed and interpreted the data; Wrote the paper.

Jawad Rasheed: Contributed reagents, materials, analysis tools or data; Analyzed and interpreted the data.

Adnan M. Abu-Mahfouz: Contributed reagents, materials, analysis tools, or data; Analyzed and interpreted the data.

## Declaration of Competing Interest

The authors declare no conflict of interest.

## Data Availability

No data was used for the research described in the article.

## References

[1] Abdollahi Mahdi, Bouyer Asgarali, Abdollahi Davoud (2016). Improved cuckoo optimization algorithm for solving systems of nonlinear equations. J. Supercomput..

[2] Tavakol Aghaei Vahid, Onat Ahmet, Eksin Ibrahim, Guzelkaya Mujde (2015). 2015 European Control Conference (ECC).

[3] Bezci Yüksel Ediz, Tavakol Aghaei Vahid, Akbulut Batuhan Ekin, Tan Deniz, Allahviranloo Tofigh, Fernandez-Gamiz Unai, Noeiaghdam Samad (2022). Classical and intelligent methods in model extraction and stabilization of a dual-axis reaction wheel pendulum: a comparative study. Results Eng..

[4] Dann Christoph, Neumann Gerhard, Peters Jan (January 2014). Policy evaluation with temporal differences: a survey and comparison. J. Mach. Learn. Res..

[5] Dorigo Marco, Birattari Mauro, Stutzle Thomas (2006). Ant colony optimization. IEEE Comput. Intell. Mag..

[6] Erol Osman K., Eksin Ibrahim (2006). A new optimization method: Big Bang–Big Crunch. Adv. Eng. Softw..

[7] Fantoni Isabelle, Lozano Rogelio (2001).

[8] Goldberg David E. (2013).

[9] Hatamlou Abdolreza (2013). Black hole: a new heuristic optimization approach for data clustering. Inf. Sci..

[10] Ben Hazem Zied, Fotuhi Mohammad Javad, Bingül Zafer (2020). Development of a Fuzzy-LQR and Fuzzy-LQG stability control for a double link rotary inverted pendulum. J. Franklin Inst..

[11] Homburger Hannes, Wirtensohn Stefan, Reuter Johannes (2022). 2022 30th Mediterranean Conference on Control and Automation (MED).

[12] Iraji Amin, Karimi Javad, Keawsawasvong Suraparb, Nehdi Moncef L. (2022). Minimum safety factor evaluation of slopes using hybrid chaotic sand cat and pattern search approach. Sustainability.

[13] Jia Shiduo, Kang Xiaoning, Cui Jinxu, Tian Bowen, Xiao Shuwen (2022). Hierarchical stochastic optimal scheduling of electric thermal hydrogen integrated energy system considering electric vehicles. Energies.

[14] Jovanovic Dijana, Marjanovic Marina, Antonijevic Milos, Zivkovic Miodrag, Budimirovic Nebojsa, Bacanin Nebojsa (2022). 2022 International Conference on Artificial Intelligence in Everything (AIE).

[15] Dervis Karaboga (2010). Artificial bee colony algorithm. Scholarpedia.

[16] Keighobadi Jafar, Hosseini-Pishrobat Mehran, Faraji Javad (2020). Adaptive neural dynamic surface control of mechanical systems using integral terminal sliding mode. Neurocomputing.

[17] Hwan Kim Jun, Oh S.J. (2000). A fuzzy PID controller for nonlinear and uncertain systems. Soft Comput..

[18] Tufan Kumbasar, Hagras Hani (2014). Big Bang–Big Crunch optimization based interval type-2 fuzzy PID cascade controller design strategy. Inf. Sci..

[19] Lewis F.L., Yesildirak A., Jagannathan Suresh (1998).

[20] Li Yiming, Wang Gencheng (2022). Sand cat swarm optimization based on stochastic variation with elite collaboration. IEEE Access.

[21] Mirjalili Seyedali (2019). Evolutionary Algorithms and Neural Networks.

[22] Mirjalili Seyedali, Lewis Andrew (2016). The whale optimization algorithm. Adv. Eng. Softw..

[23] Mirjalili Seyedali, Mohammad Mirjalili Seyed, Lewis Andrew (2014). Grey wolf optimizer. Adv. Eng. Softw..

[24] Moharam Amal, El-Hosseini Mostafa A., Ali Hesham A. (2016). Design of optimal PID controller using hybrid differential evolution and particle swarm optimization with an aging leader and challengers. Appl. Soft Comput..

[25] Montoya Oscar Danilo, Gil-Gonzalez Walter (2020). Nonlinear analysis and control of a reaction wheel pendulum: Lyapunov-based approach. Int. J. Eng. Sci. Technol..

[26] Neath Michael J., Swain Akshya K., Madawala Udaya K., Thrimawithana Duleepa J. (2014). An optimal PID controller for a bidirectional inductive power transfer system using multiobjective genetic algorithm. IEEE Trans. Power Electron..

[27] Ouyang Huimin, Hu Jinxin, Zhang Guangming, Mei Lei, Deng Xin (2019). Sliding-mode-based trajectory tracking and load sway suppression control for double-pendulum overhead cranes. IEEE Access.

[28] Price Kenneth V. (2013). Handbook of Optimization.

[29] Rahimi Afshin, Kumar Krishna Dev, Alighanbari Hekmat (2020). Fault isolation of reaction wheels for satellite attitude control. IEEE Trans. Aerosp. Electron. Syst..

[30] Rashedi Esmat, Nezamabadi-Pour Hossein, Saryazdi Saeid (2009). GSA: a gravitational search algorithm. Inf. Sci..

[31] Seyyedabbasi Amir (2022). 2022 International Conference on Theoretical and Applied Computer Science and Engineering (ICTASCE).

[32] Seyyedabbasi Amir (2022). WOASCALF: a new hybrid whale optimization algorithm based on sine cosine algorithm and levy flight to solve global optimization problems. Adv. Eng. Softw..

[33] Seyyedabbasi Amir, Kiani Farzad (2022). Sand cat swarm optimization: a nature-inspired algorithm to solve global optimization problems. Eng. Comput..

[34] Swamy Simhadri Kumara, Mohanty Banaja, Kumar Panda Sanjaya (2019). Comparative performance analysis of 2DOF state feedback controller for automatic generation control using whale optimization algorithm. Optim. Control Appl. Methods.

[35] Sun Xiaodong, Jin Zhijia, Cai Yingfeng, Yang Zebin, Chen Long (2020). Grey wolf optimization algorithm based state feedback control for a bearingless permanent magnet synchronous machine. IEEE Trans. Power Electron..

[36] Tavakol Aghaei Buse ilayda Komurcu Vahid, Saka Didar, Aydogan Bahar, Kizilca Gizem, Erener Seda (2021). 2021 29th Signal Processing and Communications Applications Conference (SIU).

[37] Tavakol Aghaei Vahid, Ağababaoğlu Arda, Yıldırım Sinan, Onat Ahmet (2022). A real-world application of Markov chain Monte Carlo method for Bayesian trajectory control of a robotic manipulator. ISA Trans..

[38] Ufnalski Bartlomiej, Kaszewski Arkadiusz, Grzesiak Lech M. (2015). Particle swarm optimization of the multioscillatory LQR for a three-phase four-wire voltage-source inverter with an *LC* output filter. IEEE Trans. Ind. Electron..

[39] Wadi Ali, Lee Jin-Hyuk, Romdhane Lotfi (2018). 2018 11th International Symposium on Mechatronics and Its Applications (ISMA).

[40] Wang Jia-Jun (2011). Simulation studies of inverted pendulum based on PID controllers. Simul. Model. Pract. Theory.

[41] Wang Jia-Jun, Kumbasar Tufan (2018). Big Bang-Big Crunch optimized hierarchical sliding-mode control of X-Z inverted pendulum. Simul. Model. Pract. Theory.

[42] Wang Jia-Jun, Kumbasar Tufan (2020). Optimal PID control of spatial inverted pendulum with Big Bang–Big Crunch optimization. IEEE/CAA J. Autom. Sin..

[43] Wang Jiao, Li Yan, Hu Gang, Yang MingShun (2022). An enhanced artificial hummingbird algorithm and its application in truss topology engineering optimization. Adv. Eng. Inform..

[44] Wolpert David H., Macready William G. (1997). No free lunch theorems for optimization. IEEE Trans. Evol. Comput..

[45] Zhang Pengfei, Wu Zhengxing, Dong Huijie, Tan Min, Yu Junzhi (2020). Reaction-wheel-based roll stabilization for a robotic fish using neural network sliding mode control. IEEE/ASME Trans. Mechatron..

